# Viewing mock crimes in virtual reality increases presence without impacting memory

**DOI:** 10.3758/s13428-024-02575-1

**Published:** 2025-02-03

**Authors:** Andrew D. Green, Andrew Clark, Melanie Pitchford, Andy Guppy

**Affiliations:** 1 School of Psychology, University of Bedfordshire, University Square, Luton, LU1 3JU UK; 2 School of Psychology, Arden University, Middlemarch Park, Coventry, CV3 4FJ UK

**Keywords:** Eyewitness memory, Virtual reality, Presence, Ecological validity

## Abstract

Traditional methods of displaying stimuli in eyewitness memory research, such as mock crime videos, are often criticised for lacking ecological validity. To overcome this, researchers have suggested using virtual reality (VR) technology to display the stimuli as it can promote a sense of presence, leading to real-world responses. However, little research has compared VR with traditional methods to demonstrate this enhanced validity. In Study 1, 54 participants viewed a mock crime video on screen or in VR while their heart rate was recorded, then completed measures of presence and emotion, and had their recall tested after 10 min. In Study 2, 74 participants’ recall was tested after a 7-day delay and included a more in-depth exploration of emotional experience. In both studies, participants in the VR group reported a statistically significant increase in their sense of general presence, spatial presence, and involvement in the scene; however, there was no statistically significant difference in recall between the groups. Participants in the VR group had a statistically significant increase in heart rate in Study 1 only, and emotional experience in Study 2 only. The findings of this research suggest that VR may provide a more ecologically valid eyewitness experience than videos, without impacting participant memory or wellbeing. The findings of the current research are discussed in relation to previous literature and implications for experimental eyewitness memory research.

**Supplementary Information:**

The online version contains supplementary material available at 10.3758/s13428-024-02575-1.

## Introduction

### Eyewitness memory research methods

Decades of research on eyewitness memory have demonstrated that eyewitnesses do not always recall the event they observed accurately. In much of this research, a participant must be presented with a to-be-remembered event. As technology has developed, so too have the methodologies for presenting to-be-remembered events in eyewitness research. Take, for example, early research on leading questions by Loftus et al. (1978), where participants were shown road traffic accidents via a series of slides. While there are a variety of methods that have been used to present eyewitness to-be-remembered stimuli such as photos (Sharps et al., 2009), written vignettes (Dornburg & McDaniel, 2006) and pre-existing media clips (LaPaglia et al., 2014), the most common method is video. Mock crime scenes can be recorded and played to participants. Another approach has been to perform the to-be-remembered event live before the participants. This article examines virtual reality (VR) as an alternative approach to presenting the to-be-remembered events in eyewitness research.

Mock crime videos offer high levels of experimental control as they can be designed and manipulated to suit the needs of the study (e.g. Takarangi et al., 2006); and are reliable as all participants see the exact same stimulus. The ability to view events with temporal realism, appropriate sound and motion may mean that this approach is more ecologically valid than other methods as the experience is more realistic than, for example, reading a written vignette. However, it cannot be ignored that participants watching a video are still in a laboratory’s relative comfort and safety, where it is challenging to elicit a level of arousal congruent to viewing a real crime (Ihlebæk et al., 2003). Real crimes may have different attentional demands on witnesses, as they may have to assess their safety or identify an escape route (Chae, 2010). Dividing attention in this way can impact memory performance (Lane, 2006), and thus recall accuracy from video stimuli may not represent real life.

Live events are one approach to overcoming the limitations of videos. Staged live events are intended to be perceived as real by participants, and thus more ecologically valid. Live events can be done with large groups of participants at one time, reducing repetition, inconsistencies, and costs. For example, Flin et al. (1992) staged a scene in a lecture where actors knocked over a projector and had an argument, with all participants viewing the event simultaneously. Presenting to a large group can be problematic as it increases the impact of any errors, such as mistakes made by actors (Wells & Penrod, 2011). Alternatively, live events can take place in small groups or individually. For example, Rubínová et al. (2021) conducted smaller eyewitness events until the required sample size was met, totalling nine repetitions. Running numerous live events can reduce consistency, as the more that are staged, the greater the opportunity for variation. Live events are also subject to unplanned factors, such as participants attempting to intervene. Thus, while live events might increase the ecological validity of eyewitness research, there are issues surrounding control and reliability.

Research directly comparing eyewitness memory of video and live stimuli has mixed findings. Ihlebæk et al. (2003) found that participants who viewed the to-be-remembered event in video format recalled more correct details than participants who viewed the event live. For example, the video group provided more details overall (*n* = 297) and was more accurate (83.8%) when describing the perpetrator’s clothing than the live group (*n* = 154; 72.7%). They argued that this was possibly due to the video offering a clear and uninterrupted view, while live participants had varied viewpoints and interacted with the perpetrators. These findings suggest that video studies may overestimate memory performance. Pozzulo et al. (2008) found no difference in eyewitness memory between a video and live group; however, there was a statistically significant increase in arousal in the live group, suggesting greater ecological validity as it elicited a greater stress response. Videos lack immersion and only elevate participant stress by a small amount compared to real life (Sauerland et al., 2016). Bates et al. (1999) found that recall was better in live event conditions (84–93% accuracy) than in video (75–88% accuracy), suggesting that this was due to the de-contextualisation of the event by portraying it on video. It was argued that videos might underestimate memory performance – a statement directly contrasting with Ihlebæk et al.’s findings.

Videos and live events are valuable methods of conducting eyewitness memory research but have limited ecological validity or reliability and control. As new technologies become available, researchers should investigate their ability to resolve these issues. One such emerging technology is virtual reality (VR), which may offer a new stimuli presentation method for eyewitness memory studies, providing both naturalistic environments and high experimental control levels (Kothgassner & Felnhofer, 2020).

### Virtual reality and presence

VR is a term that describes technologies that present a user with a computer-generated world that surrounds their senses (Slater, 2018). Environments can be presented that are computer-generated or filmed with special 360° cameras. Computer generation allows a researcher to create any imaginable environment that users can explore either with a controller or physically walking, and 360-degree videos are photorealistic, immersing a user in a video of real events in realistic environments. One argument for the use of VR in research is that it can elicit a sense of presence.

Presence, commonly described as the feeling of “being there” (Reeves, 1991), refers to the temporary blurring of the lines between the real and virtual world. Users do not believe that they have been physically drawn into the virtual world, however, they may respond and react to stimuli as if it were real (Slater, 2018). Theoretically, this means that researchers can place participants into virtual facsimiles of real situations and expect them to respond in a similar way to which they would if encountering the same situation in real life. For example, Kisker et al. (2021) found that participants experiencing presence in a virtual task walking at height would walk slower and have an increased heart rate (HR), as you would expect in real life, compared to walking at ground level. It has been found that presence in virtual environments can provoke real-world emotional (Felnhofer et al., 2015), behavioural (Kisker et al., 2021), and physiological responses (van Dammen et al., 2022).

### Eyewitness memory and VR

Eyewitness memory researchers are beginning to advocate using VR to present experimental stimuli. Like video stimuli, it can offer high levels of control, and yet still elicit reactions similar to those seen in the real world (Glomb, 2021). If participants of a VR eyewitness memory study respond to stimuli similarly to witnessing an actual crime, then an increase in presence, as well as emotional and physiological responses, could be considered an indicator of ecological validity (Kothgassner & Felnhofer, 2020; Parsons, 2015). Therefore, VR may address the methodological issues of both mock crime videos and staged live events. When comparing different stimulus presentation mediums in a non-eyewitness study, Makowski et al. (2017) found that a more emotional experience and a greater sense of presence were associated with increased memory performance. Schöne et al. (2019) compared 360 VR and 2D video stimuli and found that those in the VR condition had better recall of events than the video group. The authors suggest that events viewed in VR become part of a participant’s autobiographic memory, whereas videos do not.

Only a handful of eyewitness studies (e.g. Kim et al., 2014; Kloft et al., 2020) have presented mock crimes to participants in VR. Though presence was stated as a key factor behind the choice of using VR in these studies, it was not measured. Nyman et al. (2020) compared video and VR stimuli in a study on display method, perspective and threat level and the effects on eyewitness identification accuracy. Participants witnessed a theft either in a 360° video in VR or a normal video on a screen, and it was found that participants were more accurate in the video group. Though it was not measured, they suggested that presence measures should be taken as a manipulation check. This study compared eyewitness accuracy across video and VR stimuli, however it was specifically testing identification rather than recall of events. Identification accuracy is the ability to recognise and pick a suspect out of a lineup, whereas recall accuracy refers to the correctness of information remembered and reported about a crime. These are both important aspects of eyewitness memory, which are generally studied separately. The focus of the studies outlined in this paper is specifically on eyewitness recall accuracy.

In a recent study, Glomb et al. (2023) found that participants who viewed a mock crime in VR experienced more emotion and immersion than participants who saw the event via video, but there were no statistically significant differences in arousal or memory. Here, the authors use the term immersion as a synonym for presence, rather than the more commonly accepted definition of immersion as the objective technical properties of a system, which may elicit a psychological sense of presence (Slater, 2009). Arousal was measured by electrodermal activity. There is evidence that this measure of arousal is only weakly associated with presence, and that HR may be a more reliable measure of arousal in VR (Lee et al., 2017; Meehan et al., 2002). Glomb et al., examined free recall to measure witness memory, where participants have control over the information they provide, which is an important feature of real-life memory (Goldsmith & Koriat, 2007). However, it is possible for free recall to include only information on which the witness is highly confident and omit other details (Evans & Fisher, 2011), so it is common to follow this up with a cued recall task as they can provide additional information which may better represent their memory performance (Compo et al., 2018).

Glomb (2021) suggested that VR may be able to provide a solution to the validity and reliability issues of other eyewitness memory stimuli presentation methods, such as mock crime videos; however, little research has been done to confirm this. The current paper presents two studies that compare VR and video stimuli to see if they elicit differences in presence, physiological arousal, emotional experience, or recall. It was predicted that VR would offer a more ecologically valid experience.

## Study 1

### Background

Study 1 aimed to compare participants’ memory recall, sense of presence, emotional experience, and physiological arousal to establish if VR is an ecologically valid alternative stimulus presentation method for eyewitness memory research to video. Though inconclusive, research findings have tended to show that video stimuli may overestimate memory performance compared to similar live (Ihlebæk et al., 2003) and VR stimuli (Nyman et al., 2020), and thus it was hypothesised that participants in the video stimuli group will have significantly better recall of events than those in the VR group [H1]. As it was expected that the VR experience would be more like seeing a crime in real life compared to video, it was hypothesised that participants in the VR condition would experience a significantly higher sense of presence [H2], have a more emotional experience [H3] and that presence would mediate the relationship between emotion and memory [H4]. It was expected that a more realistic experience would increase physiological arousal, and as such it was hypothesised that there would be a significant positive correlation between presence and HR [H5] and that HR would be significantly lower in the video compared to the VR group [H6]. These hypotheses were pre-registered on the open science framework (https://osf.io/bqhnm).

### Method

#### Participants and design

The participants were 54 University of Bedfordshire staff and students (92.59% student, 38 female, 15 male, one did not disclose their gender) aged 18 to 58 years (M = 29.57, SD = 9.59). The sample size was determined via a power analysis for the comparison of the free recall between groups, using effect sizes from Schöne et al. (2019) in which the authors compared the memory of a 360° stimulus displayed on video or in VR (desired power = 0.8, alpha = 0.05, Cohen’s *d* = 0.713), which suggested a sample of 52. As a mediation analysis was planned following the findings of Makowski et al. (2017), the guidelines of Fritz and MacKinnon (2007) suggested that 54 participants would be required. First and second-year undergraduates were recruited through the SONA participant pool and received credits required for their research methods units. Participants were randomly allocated to either the video or VR group. The study hypotheses and analyses were pre-registered on the Open Science Framework (https://osf.io/bqhnm) and ethical approval was granted by the University of Bedfordshire School of Psychology Ethics Committee.

#### Materials

The to-be-remembered event was filmed using an Insta360 One 360° camera and was used for both the video and VR conditions (video version: https://osf.io/extw5; 360 VR version: https://osf.io/3v5pm). The event, lasting 2 min and 40 s, depicted a theft in the university library. A student is seen working alone and is approached by a peer, followed by a brief conversation. They both exit the room to print an assignment, leaving the victim’s items unattended on a desk. The perpetrator enters the room and steals a mobile phone, wallet and laptop before leaving again. Participants had the perspective of someone else sitting in the same area of the library. An Oculus Quest 2 VR head-mounted display was used to display the stimuli to participants in the VR group, and a 21″ computer monitor was used to display to the video group. A PowerLab 26 T and pulse transducer from ADInstruments were used to record HR from the participant’s right thumb, which was analysed using Lab Chart 7.

Visually induced motion sickness (VIMS) is a common side effect of VR use; thus, the Motion Sickness Susceptibility Questionnaire Short-form (MSSQ-Short; Golding, 1998) was used to screen for predisposition to motion sickness. The MSSQ is a widely used tool for predicting the likelihood of motion sickness, however, it largely relates to sickness induced by physical movement. As such, the Visually Induced Motion Sickness Susceptibility Questionnaire (VIMSSQ; Golding & Keshavarz, 2021) was also completed as a more specific measure of sickness experienced with electronic displays (including VR). This has been shown to predict 34% of the variance in motion sickness and increases to 56% when combined with other measures such as the MSSQ. The Simulator Sickness Questionnaire (SSQ; Kennedy et al., 1993) was used to measure participant wellbeing before and after viewing the stimulus.

The Igroup Presence Questionnaire (IPQ; Schubert et al., 2001) measured participants’ sense of presence in general, spatial presence, realness, and involvement. This scale was selected because it is similar to other presence questionnaires that can be applied to media other than VR and is relatively short. Scores for each constituent factor range from 0 to 6, and included items such as ‘How real did the virtual world seem to you?’ and ‘In the computer-generated world, I had a sense of “being there”'.

Three questions on the valence, intensity and frequency of emotion experienced were included, based on those used by Makowski et al. (2017). These questions were “What was the most prevalent emotion during the scene?”, “How intense was this emotion?” and “How frequently did you feel this emotion?” respectively. These questions were responded to on a seven-point Likert scale, have high internal consistency and can be collated into a single measure of emotional experience.

The cued recall questions were generated in a pilot study (described in the supplemental materials) following the method described by Wilford et al. (2014). Participants had unlimited time to answer the 12 questions generated, including “What colour was the victim’s hair?” and “Please describe the tattoo on the victim’s arm.” All measures were administered using the Qualtrics platform.

#### Procedure

Participants were invited to take part in a study investigating people’s memory of stimuli displayed in different mediums, though they were not informed that they would see a crime take place. They were informed that there would be memory tasks to complete, but not the specific nature of these tasks. They were randomly allocated to the video or VR group and then asked to complete the MSSQ, VIMSSQ and SSQ to screen for susceptibility to motion sickness. High scores in any of these scales would lead to the termination of the experiment for that participant; however, no participants were excluded for this reason.

Baseline HR was recorded, and participants were exposed to the mock crime video either on video or in VR. HR was recorded for the duration of the stimuli. Immediately after viewing the stimuli, participants completed the SSQ to assess their wellbeing, the IPQ to determine the level of presence and the three emotion questions before engaging in a ten-minute distractor task, a word search, to prevent rehearsal of information. Following this, they were asked to write as much information about the event witnessed as possible. There was no limit to the amount of words they could enter or the time to complete the tasks. They were prompted to provide specific details where possible. They then answered a series of cued recall questions before being thanked for their time and debriefed. Participants typed all responses to all tasks directly into the Qualtrics survey platform.

### Results and discussion

#### Ecological validity

**Fig. 1:**
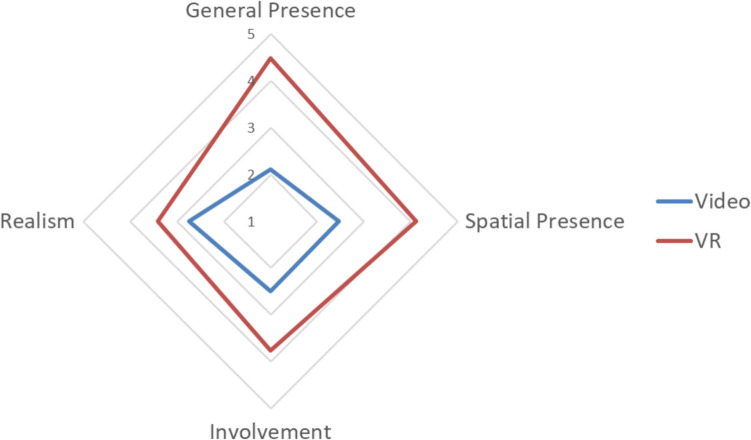
The presence profile of each group in Study 1, consisting of mean IPQ subfactor scores

Presence, HR and emotional experience were investigated as indicators of ecological validity. A one-way MANOVA was conducted to compare the groups across all four measures of presence. Using Pillai’s trace, there was a statistically significant difference between levels of presence reported between the VR and video groups, *V* = 0.41, *F*(4, 49) = 8.36, *p* < 0.001, *ηp*^2^ = 0.41. The VR group experienced a statistically significantly higher level of general presence (*p* < 0.001, *ηp*^2^ = 0.29), spatial presence (*p* < 0.001, *ηp*^2^ = 0.28) and involvement (*p* = 0.001, *ηp*^2^ = 0.18). There was no statistically significant difference between the groups in perceived realism (*p* = 0.05, *ηp*^2^ = 0.07). The authors of the IPQ (Schubert et al., 2001) suggest presenting results as a ‘presence profile’, as seen in Fig. 1. This is a radar or spider chart that presents each factor of the IPQ on its own axis, with scores connected creating a polygon. Readers can interpret each factor individually, or interpret the area of the polygon as a whole to represent the overall presence experience of an individual. Multiple conditions can be plotted on a single graph for comparison, of both factors and area. As seen in Table 1 and Fig. 1, the mean presence score was higher across all measures for the VR group. These findings suggest that viewing mock crime stimuli in VR may be more ecologically valid than viewing it on video, as participants reported a greater sense of witnessing an unmediated event [H1].

**Table 1:** Descriptive statistics for presence, heart rate and emotion across the groups in Study 1

	Video	VR
	Mean (SD)	Mean (SD)
General presence	2.11 (2.06)	4.48 (1.67)
Spatial presence	2.46 (1.48)	4.10 (1.18)
Involvement	2.50 (1.40)	3.76 (1.31)
Realism	2.73 (1.36)	3.40 (1.11)
Heart rate (BPM)	75.88 (11.12)	82.93 (12.89)
Emotional experience	4.50 (1.53)	4.91 (1.24)

An independent-samples *t* test was conducted to compare the difference in experimental HR between groups. Data from four participants, two from each group, were excluded from the analysis due to poor physiological recording. Participants in the VR group experienced a statistically significant higher HR than the video group, *t*(48) = – 2.07, *p* = 0.02, *d* = – 0.59 [H6]. This supports the notion of VR as an ecologically valid method for displaying eyewitness stimuli; however, it should be noted that no measure of previous VR experience was taken, and previous research has shown that the novelty of stimuli can affect physiological arousal (Felnhofer et al., 2015).

HR can be used as a confirmatory measure of presence, and as such, a Spearman correlation was conducted. The test indicated a statistically significant, moderate, positive correlation between HR and general presence, *r*_*s*_(48) = 0.37, *p* = 0.004 [H5].

Intensity and frequency of emotion were combined into a single emotional experience measure (α = 0.70). An independent-measures *t* test indicated that there was no statistically significant difference between the emotional experience of the two groups, *t*(52) = – 1.07, *p* = 0.14, *d* = – 0.29 [H3]. Though research has shown that VR can increase emotional experience (Estupiñán et al., 2014), our results did not show a difference in emotional experience between the two groups. This reflects the findings of previous research (Glomb et al., 2023; Makowski et al., 2017), who found no difference in emotion between a high and low immersion group. However, Tian and Zhang (2021) found that showing stimuli in VR should lead to a higher level of emotional arousal. It may be that the valence of the stimuli was not sufficiently negative or that the measure of emotion was inappropriate.

In an exploratory analysis to establish if the VR experience had a negative impact on wellbeing, the post-experiment SSQ scores of the groups were compared. A Mann–Whitney *U* test confirmed that there was no statistically significant difference in post-experiment wellbeing between the VR group (Mdn = 28.35) and the video group (Mdn = 0), *U* = 908.50, *z* = – 0.72, *p* = 0.47, *r* = – 0.08. Considering the entire sample, a Wilcoxon signed-rank test revealed that there was no statistically significant difference between the pre- (Mdn = 0, *n* = 89) and post-experiment (Mdn = 0, *n* = 89) wellbeing, *z* = – 0.04, *p* = 0.97, *r* = 0.004. This suggests that participant wellbeing did not change due to watching the stimuli and did not vary between the groups. The VR setup used in this study is no more likely to negatively impact participant wellbeing than video stimuli.

#### Recall

Participants’ freely recalled accounts were coded and scored following the guidelines from Wright and Holliday (2007). Each video was initially coded into idea units, with all possible items that a participant could recall listed. These items are categorised as person, action, object or surrounding items. For example, the perpetrator’s hair colour would be a person item. This process was completed separately by the researcher and a research assistant to ensure accuracy and completeness.

Once data had been collected, free recall was coded into idea units and compared to the previously created list. Each idea from the recall can be classed as correct, incorrect or confabulation. For example, in a scene where a man steals a phone, a free recall of “the girl stole the phone and laptop” would be scored as “the girl (incorrect person) stole (correct action) the phone (correct object) and laptop (confabulated object).” Each recall was scored by the researcher and a research assistant who was blind to the hypotheses of the study. This scoring system led to correct, incorrect and confabulation scores for each item category, which could then be summed to create total scores or analysed separately. An accuracy ratio was calculated as Ncorrect/(Ncorrect + Nincorrect + Nconfabulations). This value, ranging from 0 to 1, represents the correct items as a proportion of a participant’s entire account, with 0 being 0% accurate and 1 representing 100% accuracy. An intraclass correlation of 0.84 was found between the researcher and research assistant’s accuracy ratios, suggesting good interrater reliability. None of these were normally distributed across groups (*p* < 0.05), as participants were highly accurate, as seen in Table 2.

**Table 2:** Descriptive statistics for free and cued-recall accuracy across the groups in Study 1

		Video	VR
		Mean (SD)	Mean (SD)
Free recall	Total correct	45.74 (15.63)	47.19 (15.53)
	Total incorrect	1.26 (1.53)	2.22 (2.12)
	Total confabulation	.22 (.51)	.11 (.32)
	Accuracy ratio	.97 (.03)	.96 (.04)
Cued recall	Total correct	7.96 (1.16)	7.41 (2.10)
	Total incorrect	2.85 (1.61)	3.52 (1.65)
	Total “Not Sure”	1.19 (1.14)	1.07 (1.36)
	Accuracy ratio	.74 (.13)	.67 (.17)

Cued recall responses were scored as either correct, incorrect or a “not sure” response. A cued recall accuracy ratio was calculated as Ncorrect/(Ncorrect + Nincorrect). Confabulations were not present in the cued recall data due to the nature of the questions. “Not sure” responses were not treated as incorrect but rather removed from the accuracy calculation. A cued-recall accuracy ratio can be directly interpreted as a percentage of accuracy as with the free recall. Total correct items, “not sure” and the cued accuracy ratio were not normally distributed (*p* < 0.05). Total correct responses, and thus the accuracy ratio, were generally high.

A maximum of 322 units of information were available to be freely recalled, and there were 12 cued recall questions. As can be seen from the accuracy ratios in Table 2, participants in the VR group had lower accuracy ratios in the free and cued recall tasks than the video group. VR participants, on average, reported more correct and incorrect items in the free recall task, and more incorrect items in the cued task. A one-way MANOVA was conducted to compare the free recall scores of the two groups. Using Pillai’s trace, there was no statistically significant difference between the groups, *V* = 0.15, *F*(4, 49) = 2.18, *p* = 0.09, *ηp*^2^ = 0.15 [H1]. A second one-way MANOVA was conducted to compare cued recall performance across the groups. Using Pillai’s trace, there was no statistically significant difference between the groups, *V* = 0.08, *F*(3, 50) = 1.48, *p* = 0.23, *ηp*^2^ = 0.08 [H1].

Mediation analysis was planned to determine if the relationship between emotional experience and recall is mediated by the sense of presence, as seen in Makowski et al. (2017). However, emotional experience did not significantly correlate with free (*r*_*s*_(52) = – 0.12, *p* = 0.20) or cued (*r*_*s*_(52) = – 0.16, *p* = 0.13) recall accuracy ratios and thus there was no evidence of a relationship between these variables [H4].

The findings of the first study are similar to those of Glomb et al. (2023) who found that VR eyewitness stimuli elicit a greater sense of presence than a video, and there was no difference in recall. It may be that an increased sample is needed, as a post hoc power analysis suggested that at least 74 participants are required to achieve the desired power. However, the authors chose to stop data collection in line with the preregistration, and focus on resolving the limitations for Study 2. As recall was generally very accurate, it may be that the stimulus was not complex enough and the 10-min delay was too short. Flowe et al. (2018) found that the average retention interval of real cases was much longer than those used in laboratory studies (approximately 7–8 days). Meissner (2002) found that participants’ recall was less accurate and complete after a 1-week delay than immediate recall. This finding was confirmed in an archival study by van Koppen and Lochun (1997) who found that witnesses of real crimes reported a less complete description after a longer retention period. Unlike Glomb et al. (2023), Study 1 showed increased physiological arousal, likely due to the choice of measure but no difference in emotional experience.

Increases in presence and HR suggest that VR may be a more ecologically valid stimuli presentation method for eyewitness memory research than video; however, the novelty of VR use was not accounted for. Using 360-degree videos in VR had no reported negative impact on participants’ wellbeing and afforded a sense of location and action within the stimuli event. There was no statistically significant difference in participant emotional experience, which is perhaps due to the stimulus content or measures used. Though there was no statistically significant difference in recall, there is some evidence to suggest that the VR group may have been less accurate, which may be more clearly demonstrated with a longer delay before recall.

## Study 2

### Background

Study 2 was designed to address the limitations of Study 1. A measure of VR experience was included to ensure that changes in arousal were not due to the novelty of VR (Felnhofer et al., 2015). Two different emotion measures were chosen, addressing both broad dimensions and specific emotions to better understand the participants’ emotional experiences. The study was designed with a larger delay between stimuli and recall to better represent real-life witness experiences. The stimulus was designed to be more emotional, as the event in study one may have lacked clear negative valence.

As in Study 1, it was hypothesised that the video group would have a significantly better recall of events than the VR group [H1]. Due to the expected realism of VR, it was hypothesised that the VR group would experience a significantly greater sense of presence [H2], have a more emotional experience [H3], that there would be a positive correlation between presence and HR [H4] and that the video group would have a significantly lower HR than the VR group. These hypotheses were pre-registered on the open science framework (https://osf.io/t5r9h).

### Method

#### Participants and design

A sample size of 74 participants was determined following a power analysis for the comparison of the free recall between groups, using data from the equivalent analysis in study 1 (desired power = 0.8, α = 0.05, V = 0.15). As data were collected at two time points, it was decided to collect data until the required number had completed all elements of the study. Overall, 89 students and staff from the University of Bedfordshire (78.65% student, 57 female, 29 male, three other/did not disclose) aged 18 to 67 years (M = 31.36, SD = 10.97) took part. Participants were randomly allocated to either the video or VR group. The study hypotheses and analyses were pre-registered on the Open Science Framework (https://osf.io/t5r9h) and ethical approval was granted by the University of Bedfordshire School of Psychology Ethics Committee.

#### Materials

The to-be-remembered event was filmed using an Insta360 OneX2, 360-degree camera and was used for both the video and VR conditions (video version: https://osf.io/fgu48; 360 version: https://osf.io/w2hbn). The event, which lasted 2 min and 9 s, depicted four students sitting in a cafe, either reading or using their phones. One of them strikes up a conversation with the first victim, and discretely steals their phone. As they exit the scene, they take the opportunity to steal a second phone when the second victim is distracted. Once the perpetrator has exited the scene, the second victim realises what has happened and questions the others. The victim becomes agitated and blames one of the others before exiting the scene to report the incident. The scenario in Study 1 had no interaction between victim and perpetrator, and the emotional consequences were not seen. This stimulus used in Study 2 was specifically designed to be more emotive by showing the victims’ emotional response to the situation. The same pilot procedure was used from Study 1 to generate 18 cued recall questions, including “What was the male victim doing when his belongings were stolen?” and “Please describe the perpetrator’s hair”. An HTC Vive Pro 2 VR head-mounted display was used to present the stimuli to participants in the VR group, and a 21″ computer monitor was used to present to the video group. HR was recorded in the same way as Study 1, using a PowerLab 26 T and pulse transducer attached to participant’s right thumb. Previous VR experience was measured using two questions from Saffo et al. (2021). These questions were “How often do you use VR” and “How often do you play video games, both in VR and otherwise?” and responses ranged from never to once per day on a five-point Likert scale.

In Study 1, there were no issues of VIMS with participants, so the MSSQ (Golding, 1998) was removed, as it did not help predict participant wellbeing. However, the VIMSSQ (Golding & Keshavarz, 2021) and SSQ (Kennedy et al., 1993) were still used. The IPQ (Schubert et al., 2001) was used again to measure presence.

The Affective Slider (AS; Betella & Verschure, 2016) was used to measure overall valence and self-reported arousal using a slider that runs from 0 (negative emotion/low arousal) to 1 (positive emotion/high arousal), with 100 steps in between. The Positive and Negative Affect Schedule (PANAS; Watson et al., 1988) is a 20-item self-report scale that was used to measure positive and negative affect, as well as specific emotions experienced, such as guilt, fear, and enthusiasm. All measures were administered using the Qualtrics platform.

#### Procedure

Participants were invited to participate in a study investigating people’s memory and experiences of stimuli displayed in different mediums. As in Study 1, they were not informed about the crime event specifically. They were also informed that there would be memory tasks to complete, but not the specific nature of these tasks. Participants were screened with the VIMSSQ and SSQ, however, as the VR stimulus did not involve the simulated movement of the participant, there were no withdrawals due to potential sickness.

Baseline HR was recorded for 1 min before participants were randomly allocated to a group. Participants then viewed the mock crime stimulus on video or in VR while their HR was recorded. Subsequently, all participants completed the IPQ to assess their sense of presence and the AS and PANAS to measure their emotional experience.

One week later, participants were asked to complete a free and cued recall task with the same instructions as Study 1. Participants typed all responses to all tasks directly into the Qualtrics survey platform. Upon completion of the data collection, participants could enter a prize draw for a £50 Amazon voucher.

### Results and discussion

#### Ecological validity

**Fig. 2:**
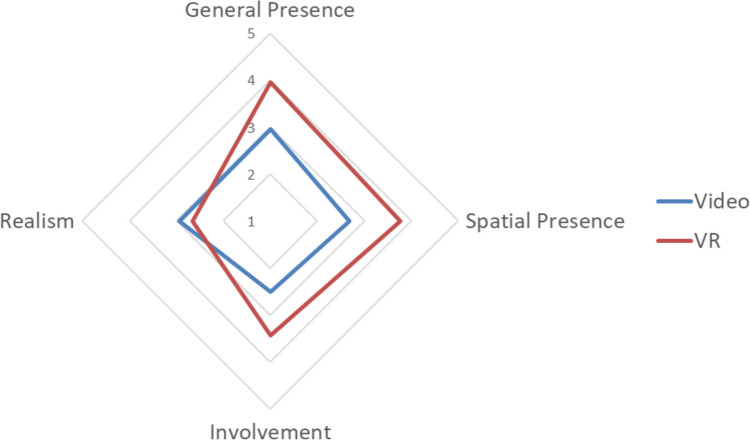
The presence profile of each group in Study 2, consisting of mean IPQ subfactor scores

As with Study 1, presence, HR and emotional experience were investigated as indicators of ecological validity. A one-way MANOVA was conducted to compare the four IPQ presence measures across the groups. Using Pillai’s trace, there was a statistically significant difference evident between levels of presence reported between the groups, *V* = 0.24, *F*(4, 84) = 6.46, *p* < 0.001, *ηp*^2^ = 0.24. The VR group experienced a statistically significantly higher level of general presence (*p* = 0.009, *ηp*^2^ = 0.08), spatial presence (*p* < 0.001, *ηp*^2^ = 0.15) and involvement (*p* = 0.001, *ηp*^2^ = 0.12). The VR group experienced a greater level of presence in each case, as can be seen in Table 3 and Fig. 2. There was no statistically significant difference between the groups in perceived realism (*p* = 0.09, *ηp*^2^ = 0.03). This pattern of results is similar to those found in Study 1 and supports the notion that the VR group had a more ecologically valid experience [H2].

**Table 3:** Descriptive statistics for presence, heart rate and emotion across the groups in Study 2

	Video	VR
	Mean (SD)	Mean (SD)
General presence	2.96 (1.77)	3.95 (1.75)
Spatial presence	2.68 (1.30)	3.77 (1.30)
Involvement	2.51 (1.23)	3.44 (1.27)
Realism	2.94 (.75)	2.66 (.77)
Heart rate (BPM)	77.37 (10.81)	78.59 (12.26)
AS Arousal	64.62 (26.89)	75.82 (19.12)
AS Valence	54.71 (23.78)	74.00 (20.56)
PANAS PA	21.29 (6.38)	30.21 (9.39)
PANAS NA	14.27 (5.97)	13.18 (3.99)

An independent-samples *t* test was conducted to compare the difference in experimental HR between groups. Data from five participants were excluded from the analysis due to poor physiological recording (four from the video group). There was no statistically significant difference in HR between the two groups, *t*(82) = – 0.48, *p* = 0.32, *d* = – 0.11 [H5]. HR can be used as a confirmatory measure of presence, and as such, a Spearman correlation was conducted, which indicated that there was not a statistically significant relationship between HR and general presence, *r*_*s*_(82) = – 0.06, *p* = 0.29 [H4]. These findings are in direct contrast to Study 1, where the VR group had a higher HR and there was a positive correlation between HR and presence.

**Fig. 3:**
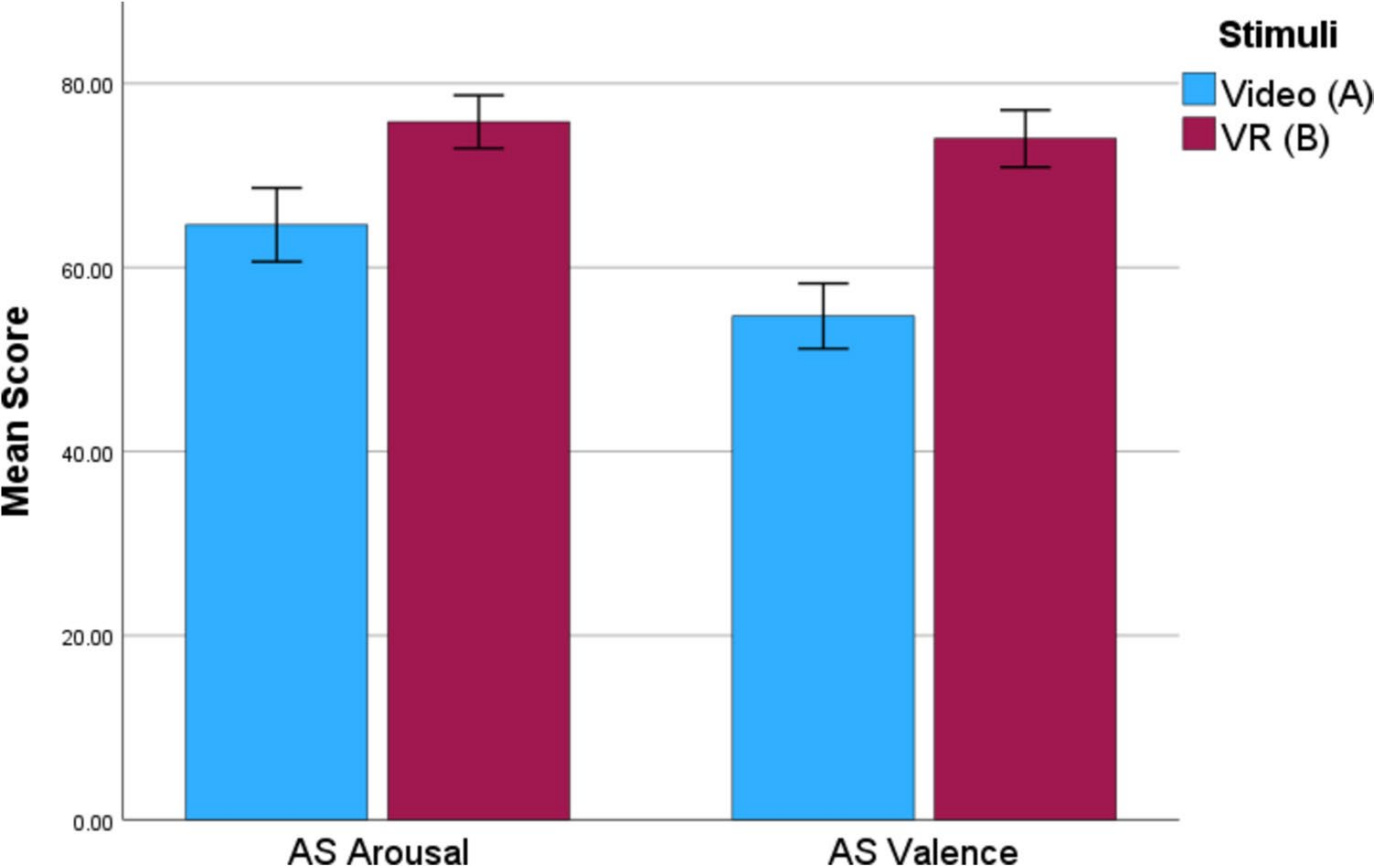
Mean AS scores across groups in Study 2. Error bars ± 1 standard error

**Fig. 4:**
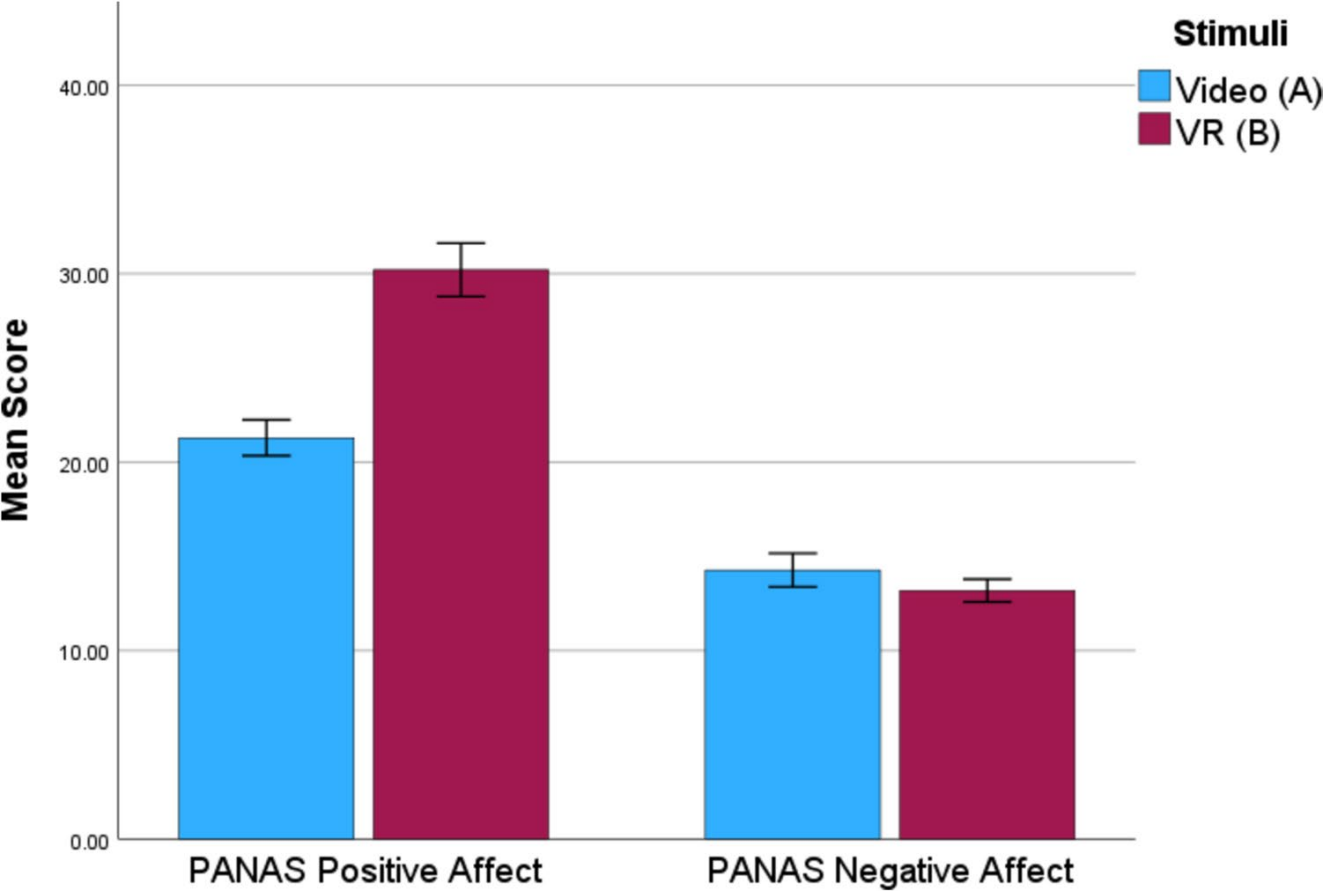
Mean PANAS scores across groups in Study 2. Error bars ± 1 standard error

A one-way MANOVA was conducted to compare the emotional experience of the two groups, as measured by the AS and PANAS. Using Pillai’s trace, there was a statistically significant difference between the emotional experience of the groups, *V* = 0.30, *F*(4, 84) = 8.93, *p* < 0.001, *ηp*^2^ = 0.30. Univariate comparison of the emotion measured revealed that there was a statistically significant difference in AS arousal (*p* = 0.03, *ηp*^2^ = 0.06), AS valence (*p* < 0.001, *ηp*^2^ = 0.16) and PANAS positive affect (PA) scores (*p* < 0.001, *ηp*^2^ = 0.24), with the VR group scoring higher in each instance as illustrated in Figs. 3 and 4 [H3]. There was no statistically significant difference in PANAS negative affect (NA) scores (*p* = 0.32, *ηp*^2^ = 0.01). To ensure that this effect was not caused by the novelty of VR use, a one-way ANCOVA was conducted to compare these scores between the two groups while controlling for VR experience. The results showed that there was still a statistically significant difference between the groups AS arousal score [*F*(1,86) = 5.04, *p* = 0.03], AS valence score [*F*(1,86) = 16.61, *p* < 0.001], and PANAS positive affect [*F*(1,86) = 27.23, *p* < 0.001] suggesting that novelty was not the cause of the increased emotional experience.

To explore this further, each item in the positive affect scale of the PANAS was compared between participants with a MANOVA. Using Pillai’s trace, there was a statistically significant difference evident between the groups’ scores on the positive affect items of the PANAS, *V* = 0.38, *F*(10, 78) = 4.85, *p* < 0.001, *ηp*^2^ = 0.38. Participants in the VR group reported statistically significantly higher scores on all items except for “strong” and “attentive”, as seen in Table 4.

**Table 4:** PANAS PA item scores by group

	Video	VR		
	Mean (SD)	Mean (SD)	*P*	*ηp*^2^
Interested	3.53 (1.10)	4.09 (1.03)	.02	.07
Excited	1.8 (.99)	3.20 (3.20)	< .001	.27
Strong	1.64 (1.04)	2.11 (1.35)	.07	.04
Enthusiastic	1.71 (.92)	3.09 (1.40)	< .001	.26
Proud	1.22 (.64)	2.02 (1.27)	< .001	.14
Alert	3.18 (1.15)	3.84 (1.14)	.008	.08
Inspired	1.40 (1.03)	2.59 (1.45)	< .001	.19
Determined	1.60 (1.12)	2.64 (1.62)	.001	.13
Attentive	3.09 (1.40)	3.66 (1.31)	.05	.04
Active	2.11 (1.28)	2.95 (1.51)	.006	.09

Though the VR group did not experience an increase in physiological arousal, there was an increase in self-reported emotional arousal. Interestingly, participants in the VR group reported a greater level of pleasure with the AS and more positive emotions in the PANAS; whereas it might be expected that if a participant were to respond as if a crime were really taking place, then they would experience more negative emotions. However, the PANAS NA showed no difference between the groups.

#### Recall

Free and cued accuracy ratios were calculated as in Study 1. An intraclass correlation of 0.70 was found between the researcher and research assistant’s free recall accuracy ratios, suggesting moderate interrater reliability. A maximum of 513 units of information were available to be freely recalled, and there were 18 cued recall questions. As seen in Table 5, participants in both groups were highly accurate in the free recall task, and less so in the cued recall. The mean accuracy ratios for these are very similar across groups. A one-way MANOVA was conducted to compare the free recall scores of the two groups. Using Pillai’s trace, there was no statistically significant difference between the groups, *V* = 0.04, *F*(4, 69) = 0.63, *p* = 0.64, *ηp*^2^ = 0.04 [H1]. A second one-way MANOVA was conducted to compare cued recall performance across the groups. Using Pillai’s trace, there was no statistically significant difference between the groups, *V* = 0.03, *F*(3, 74) = 1.48.80, *p* = 0.50, *ηp*^2^ = 0.03 [H1]. The findings show no statistically significant differences in recall between the two groups, which is similar to the findings of some previous literature (Buttussi & Chittaro, 2018; Makowski et al., 2017) as well as Study 1. Though the VR experience could be considered more ecologically valid in terms of presence, there was no statistically significant difference in arousal or negative emotion, which can impact memory (Bowen et al., 2018; Marr et al., 2021; Pezdek et al., 2021).

**Table 5:** Descriptive statistics for free and cued recall accuracy across the groups in Study 2

		Video	VR
		Mean (SD)	Mean (SD)
Free recall	Total correct	28 (14.73)	29.11 (13.36)
	Total incorrect	1.49 (1.43)	1.43 (1.26)
	Total confabulation	1.86 (1.97)	1.97 (1.76)
	Accuracy ratio	.89 (.08)	.90 (.07)
Cued recall	Total correct	8.51 (2,69)	7.86 (3.15)
	Total incorrect	6.46 (2.28)	6.27 (3.25)
	Total “not sure”	3.02 (2.70)	3.86 (3.66)
	Accuracy ratio	.57 (.14)	.56 (.19)

In an exploratory analysis to establish if the VR experience had a negative impact on wellbeing, the post-experiment SSQ score of the groups was compared. A Mann–Whitney *U* test confirmed that there was no statistically significant difference in post-experiment wellbeing between the VR group (Mdn = 28.35) and the video group (Mdn = 0), *U* = 908.50, *z* = – 0.72, *p* = 0.47, *r* = – 0.08. As with Study 1, this finding suggests that the specific VR set-up used in this experiment had no negative impact on participant wellbeing, and can be considered a relatively safe alternative to videos.

Study 2 further supports the ecological validity of VR as a stimuli display method in eyewitness memory research, as participants reported an increased sense of presence and a more emotional experience when viewing the mock crime in VR. However, the lack of physiological arousal and the valence of the emotions warrant further investigation.

## General discussion

The experiments described in this article suggest that viewing a mock crime in VR may be a more ecologically valid alternative to traditional videos in eyewitness memory research. Participants in the VR groups reported a higher experience of presence in the event they viewed compared to the video groups. Participants in the VR groups had a stronger feeling of being and acting in a real, unmediated environment, and their attention was focused more on the crime stimulus than the real world compared to participants in the video groups. These findings are similar to previous research that has demonstrated that virtual environments elicit a greater sense of presence than screens (Baptie et al., 2021; Ochs & Sonderegger, 2022), suggesting that the VR group had a more ecologically valid witness experience than the video group (Kothgassner & Felnhofer, 2020; Parsons, 2015).

Witnessing a crime can be stressful, something which can be challenging to replicate in the laboratory (Ihlebæk et al., 2003). However, VR can elicit real-world physiological responses (Kisker et al., 2021). Our findings in Study 1 support this, as the VR group had a higher HR, and there was a positive correlation between presence and HR. The more someone felt that they were “there” within the crime event the higher their HR was, supporting the ecological validity of this research method. To date, this is the first eyewitness memory study demonstrating a significant difference in arousal response between a video and VR group. However, this was not the case in Study 2, where there was no statistically significant difference in HR between the groups or a correlation with presence. This is in line with the findings of Glomb et al. (2023) who found no differences in arousal between video and VR witness participants. Their stimuli, as with Study 2, included social interaction which they explained may have made it more pleasant to view. It may also be that the stimulus event was not arousing enough. Nevertheless, participants in the VR group self-reported a higher level of arousal. It has been seen in previous VR research that increases self-reported psychological arousal do not necessarily translate to cardiovascular changes (Marin-Morales et al., 2021).

There was no statistically significant difference between the groups with the feeling of realism, comparing the virtual world to the real one. This may be due to image resolution, which is lowered when filming in 360 degrees. Research has shown that the level of visual realism can impact both arousal responses (Slater et al., 2009a, b) and IPQ realism scores (Weiß et al., 2021). Though the image quality did not negatively affect the other dimensions of presence reported, future researchers in the area should strive to use the highest possible resolution 360° video cameras. Our findings regarding participants’ emotional experiences are inconclusive. Study 1 used an emotion measure from a similarly designed study (Makowski et al., 2017) and found no differences between the video and VR groups. It may be that this measure of emotion was not sensitive enough to discern existing differences. This is supported by Study 2, as when the PANAS used, there was a statistically significant difference in emotional experience. Participants in the VR group had a higher self-reported emotional arousal, found the event to be more pleasurable and experienced positive emotions more strongly than the video group, though there was no difference in negative emotions. If the VR group experienced the event as more realistic, then it is counterintuitive for the emotions to be more positive when viewing a crime. Analysis of covariance confirmed that these differences in positive emotion were not due to the novelty of using VR technology. Perhaps despite the intended negative nature of the experience, VR is more enjoyable due to the immersive nature of the technology (Glomb et al., 2023). VR participants rated the experience as more pleasurable and that they were more interested, excited and enthusiastic, which would support this idea. Other research, however, has successfully elicited negative emotions in VR (Felnhofer et al., 2015; Riva et al., 2007).

Free recall accuracy was high in both groups and in both studies. Eyewitness memory can be accurate and reliable under optimal conditions, though it is highly susceptible to contamination (Wixted et al., 2018) and optimal conditions may be difficult to achieve (Wade et al., 2018). It is likely that free recall was so highly accurate due to participants omitting details in which they were less confident (Evans & Fisher, 2011). The cued recall findings support this idea, as when pressed to answer specific questions participant accuracy was much lower. While we had predicted that VR would have a negative impact on recall, we did not find this to be the case. Our predictions were made before the publication of Glomb et al. (2023) who also found that VR did not negatively impact free recall compared to a video condition. Our findings go further than Glomb et al. in that we have demonstrated that VR does not improve recall compared to video, despite the increase in positive effect, which can enhance memory (Mancuso et al., 2023). Our findings also suggest that VR does not negatively impact recall compared to video. This means that VR could be an appropriate alternative to video stimuli for eyewitness memory experiments. However, as previous literature suggests, video research may overestimate participant memory compared to real life (Ihlebæk et al., 2003), and thus, if we consider VR as truly ecologically valid, we might expect to see a lower recall accuracy. The 360° stimuli used in the VR groups for both studies were still in essence videos, and although participants could change their view by turning their head, it was still a relatively static position, which may explain the similar memory results.

### Limitations and future directions

The ability of the stimuli to elicit predicted real-world responses lacked consistency. In Study 1, VR participants experienced increased physiological arousal but did not report changes in emotion; the opposite was found in Study 2. The lack of difference in HR in Study 2 supports the notion that it is difficult to elicit real-world stress responses in eyewitness memory research (Chae, 2010; Ihlebæk et al., 2003) and suggests that further research is needed to clarify the extent to which VR can prompt real-world physiological responses in the context of eyewitness research. The lack of emotional difference in Study 1 is likely due to the choice of measure, and the authors suggest the PANAS and AS for future research in the area. In Study 2, participants in the VR group reported more positive emotions, possibly due to the inherently enjoyable nature of using VR. However, VR has previously been used to successfully elicit negative emotions (Felnhofer et al., 2015; Riva et al., 2007), so it may be that the stimuli did not have a clear enough negative valence. Gall et al. (2021) also suggest that the illusion of embodiment in a virtual body can increase the intensity of emotions experienced in VR. Due to the use of 360° videos in these studies, embodiment in this way was not feasible. However, future eyewitness studies should test a computer-generated VR environment in which a user can embody an avatar and possibly experience emotions more naturally.

The main limitation of this study is that while it suggests VR may provide a more ecologically valid witness experience, it does not have a real-world comparison. Future studies should include a live event for comparison with the video and VR groups, allowing for more definitive conclusions to be drawn. Knowing the real-world recall accuracy, emotional experience and physiological response to the stimuli would allow researchers to determine if VR is a better facsimile for real-life events than videos.

### Conclusion

Despite the lack of a real-world comparison group, there is still evidence that VR stimuli may offer a more ecologically valid witness experience than a traditional video. Participants viewing a mock crime in VR reported feeling a greater sense of “being there” within the event and paid more attention to the virtual world than the laboratory around them. There is also some evidence that VR can provide a more emotional and physiologically arousing experience, which does not positively or negatively impact memory compared to watching a video. Using a 360° video in VR led to no cases of VIMS or discomfort in participants, who reported having a pleasant experience. This type of VR stimulus was simple to record and display to participants and was only a little more time-consuming. VR hardware may require extra technical expertise, though consumer products such as those used in this study are relatively easy to use. More research is needed to validate this methodology with the inclusion of a live group, computer-generated environments with avatars, and the use of other extended reality technologies such as augmented reality.

Due to the applied nature of eyewitness memory research, the realism of participant experience and response should be a primary concern, and as such, it is suggested that future researchers consider using VR. The studies described here specifically used VR stimuli when examining eyewitness recall of events, and it would be interesting to see the same techniques validated for eyewitness identification research.

## Supplementary Information

Below is the link to the electronic supplementary material.

Supplementary file1 (DOCX 13 KB)

## Data Availability

The stimuli generated and used for these studies, as well as the datasets generated and coding methods, are available on the Open Science Framework. Study 1 stimulus: https://osf.io/extw5 (360 version: https://osf.io/3v5pm). Study 1 data: https://osf.io/p5rbs Study 2 stimulus: https://osf.io/fgu48 (360 version: https://osf.io/w2hbn). Study 2 data: https://osf.io/2byqn Supplementary materials: https://osf.io/7y69f

## Declarations

### Open practices

Both studies were preregistered using the Open Science Framework (Study 1: https://osf.io/bqhnm Study 2: https://osf.io/t5r9h).
