# Supplementary material for: Viewing mock crimes in virtual reality increases presence without impacting memory
Source: Behav Res Methods. 2025 Feb 3;57(3):88. doi: 10.3758/s13428-024-02575-1 (PMC11790719; doi:10.3758/s13428-024-02575-1)
Supplement: Supplementary file 1 — Supplementary file1 (DOCX 13 KB) [file 13428_2024_2575_MOESM1_ESM.docx]

**Supplemental Materials**

Pilot study to generate cued recall questions

Once each stimulus video was created, a pilot study was conducted to develop cued recall questions. Participants viewed the video on a computer screen and were asked to provide a written free recall of events, remembering as much detail as possible from the video. Following the coding method described by Wilford et al. (2014), the narrative was coded into idea units. These units are small phrases that convey meaningful ideas expressed by the participants, such as “the victim entered the room”. Each participant’s account coded into these idea units by the researcher and a second coder separately. Idea units which few participants recalled were labelled as non-frequent (peripheral) items, moderately well remembered we labelled as medium (neutral) items, and well-remembered as frequent (central) items.

In study 1, 12 idea units were selected to be developed into cued recall questions: four peripheral, four neutral and four central. For example, the unit “the victim had a bee tattoo on his arm” because “what was the large tattoo on the victims arm?”. Following the creation of the cued recall questions, a few further participants were shown the video and asked to complete the cued recall task to ensure that each question was clear and possible to answer. Each question was answered correctly at least once, suggesting that they were appropriate for use in the main study. This same process was followed for Study 2, though due to the more complex nature of the scene and volume of information freely recalled by pilot participants, 18 questions were developed (six of each type).
